# The Anti-Inflammatory Effect of Novel Antidiabetic Agents

**DOI:** 10.3390/life12111829

**Published:** 2022-11-09

**Authors:** Panagiotis Theofilis, Marios Sagris, Evangelos Oikonomou, Alexios S. Antonopoulos, Gerasimos Siasos, Kostas Tsioufis, Dimitris Tousoulis

**Affiliations:** 1 1st Cardiology Department, “Hippokration” General Hospital, University of Athens Medical School, 11527 Athens, Greece; 2 3rd Cardiology Department, Thoracic Diseases Hospital “Sotiria”, University of Athens Medical School, 11527 Athens, Greece

**Keywords:** diabetes mellitus, inflammation, SGLT-2 inhibitor, GLP-1 receptor agonist, DPP4 inhibitors

## Abstract

The incidence of type 2 diabetes (T2DM) has been increasing worldwide and remains one of the leading causes of atherosclerotic disease. Several antidiabetic agents have been introduced in trying to regulate glucose control levels with different mechanisms of action. These agents, and sodium-glucose cotransporter-2 inhibitors in particular, have been endorsed by contemporary guidelines in patients with or without T2DM. Their widespread usage during the last three decades has raised awareness in the scientific community concerning their pleiotropic mechanisms of action, including their putative anti-inflammatory effect. In this review, we delve into the anti-inflammatory role and mechanism of the existing antidiabetic agents in the cardiovascular system and their potential use in other chronic sterile inflammatory conditions.

## 1. Introduction

Diabetes mellitus type 2 (T2DM) is a major pandemic in Western societies, with serious consequences such as chronic kidney disease (CKD), atherosclerosis, and heart failure. Traditional therapeutic approaches have tried to improve hyperglycemia, with evidence indicating no links to the prevalence of complications. As a result, recent breakthroughs in T2DM pharmacological therapy point to the reversal of the complicated pathophysiology rather than merely dysglycemia. In this context, novel antidiabetic medications have shown substantial efficacy in improving cardiorenal outcomes, according to the landmark randomized clinical trial results.

Low-grade sterile inflammation has been identified as a significant risk factor for cardiorenal diseases [1,2,3,4,5,6]. It is also necessary to emphasize the effect of established cardiovascular risk factors in increasing inflammation [7,8,9]. Moreover, inflammation plays an important role in the pathogenesis of T2DM [10]. Therefore, in this review article, we highlight the deleterious effect of inflammation in T2DM pathophysiology and complications, while we also elaborate on the evidence concerning the anti-inflammatory potential of novel antidiabetic medication, namely, dipeptidyl peptidase-4 inhibitors (DPP4is), glucagon-like peptide-1 (GLP-1) receptor agonists, and sodium-glucose cotransporter-2 (SGLT2) inhibitors.

## 2. Inflammation as a Pathophysiologic Mechanism in DM and Its Complications

The inflammatory hypothesis of T2DM has been evaluated in several human studies, which have assessed the relationship between DM and inflammatory biomarkers. In a case–control study of 2333 subjects, the presence of T2DM was associated with elevated high-sensitivity C-reactive protein (hsCRP) in both male and female participants [11]. The significance of this relationship remained unaffected even after adjustment for multiple confounders. Body mass index, waist circumference, HbA1c, fasting insulin, and C peptide were the factors influencing hsCRP levels in diabetic subjects [11]. A correlation between HbA1c and hsCRP was observed in T2DM subjects irrespective of coronary artery disease presence, as shown by Bahceci et al. [12]. Moreover, TNF-α was positively correlated with HbA1c and insulin resistance, measured by the Homeostatic Model Assessment for Insulin Resistance index, in subjects with DM [13]. 

Multiple inflammatory pathways are involved in the pathophysiology of DM. First and foremost, β cell dysfunction may be regulated by the nuclear factor-κB (NF-κB) signaling pathways. Proinflammatory molecules, including cytokines and pathogen- or damage-associated molecular patterns are able to activate NF-κB through activation of the inhibitory κB protein (IκB) kinase (IKK) complex [14,15]. In the noncanonical NF-κB induction, the TNF receptor-associated factor 2 (TRAF2) recruits cellular inhibitors of apoptosis 1 and 2 (cIAP1/2), leading to TRAF3 proteolysis and NF-κB-inducing kinase (NIK) accumulation. Consequently, NIK phosphorylates IKKα, causing the inhibitory protein p100 to be processed into the active component p52, which binds to RelB and translocates to the nucleus to promote gene expression [16]. However, the deleterious effect of the noncanonical pathway and its ability to promote DM has been characterized by controversial evidence [17,18,19].

The emerging role of nucleotide-binding oligomerization domain and leucine-rich repeat pyrin 3 domain (NLRP3) inflammasome in DM ought to be stressed. Its activation is a two-step process consisting of initial “priming” with the aid of NF-κB-mediated cytokine transcription [20] and subsequent activation mediated by lysosomal instability, mitochondrial damage, and potassium efflux [20]. Mitochondrial injury is of great importance since it can promote NLRP3 inflammasome activation in multiple ways, such as the release of mitochondrial reactive oxygen species, DNA, and cardiolipin, which is an activator of NLRP3 [20]. Impaired autophagy, the vital process of lysosomal clearance of cellular debris, may constitute another factor aiding NLRP3 activation [21]. The inflammasome assembly occurs after NLRP3 activation, and the pro-IL-1 and pro-IL-18 are cleaved into IL-1 and IL-18.

Alterations in the gut microbiome is a novel mechanism that may lead to DM via several pathways, including the augmentation of inflammation. It is known that several intestinal bacteria modulate inflammation. Among the bacteria known to possess anti-inflammatory actions are *Roseburia intestinalis* [22], *Bacteroides fragilis* [23], *Akkermansia muciniphila* [24], *Lactobacillus plantarum* [25], and *Lactobacillus casei* [26], by promoting IL-10 production, thus preventing ageing-related insulin resistance [27]. Moreover, Lactobacilli could provide anti-inflammatory effects by regulating the expression of proinflammatory cytokines [28]. Moreover, butyrate-producing bacteria such as *Roseburia* spp. and *Faecalibactrium* spp. may inhibit NF-κB activity [29]. According to this evidence, we can assume that the lack of such bacterial strains in the gut environment may play an important role in the constitution of a proinflammatory state and, ultimately, the development of DM. Additionally, the abundance of other strains such as *Fusobacterium nucleatum* and *Ruminococcus gnavus* leads to proinflammatory cytokine overexpression [30,31].

Inflammation plays a detrimental role in the development of diabetic complications, most notably atherosclerosis and kidney injury. Beginning with atherosclerosis, inflammation has emerged as a critical mechanistic component [1,32]. DM, through inflammatory stimuli, is an important regulator of endothelial dysfunction, which is among the initial steps of atherogenesis [33]. As a result of hyperglycemia, the overexpression of advanced glycation end products (AGEs) and their receptors (RAGE) disrupts nitric oxide bioavailability and promotes reactive oxygen species production from the vascular endothelial cells [33]. Moreover, hyperglycemia-induced overexpression of AGEs and reactive oxygen species promotes monocyte recruitment, low-density lipoprotein oxidation, and, ultimately, foam cell formation [34]. 

Renal inflammation is among the main pathophysiologic mechanisms involved in the development of diabetic kidney disease. This inflammatory state is propagated by high glucose levels and AGEs [35], with ensuing release of damage-associated molecular patterns and activation of pattern recognition receptors. It has been previously proven through kidney biopsies that macrophages invade glomeruli and renal interstitial space [36], as a result of bone marrow-derived monocyte recruitment and differentiation in the setting of renal injury [37]. The degree of macrophage accumulation has also been associated with the progression to end-stage kidney disease (ESKD) [38]. Other than macrophages, the renal concentration of activated T cells is significantly higher in patients with T2DM and is attributable to proteinuria [39]. Apart from the increased expression of well-established inflammatory mediators such as IL-6 [40], the upregulation of complement C3, CXC-chemokine ligand 6 (CXCL6), and CC-chemokine ligand 2 (CCL2) has been observed in kidney biopsy samples [41]. Concerning human studies, increased high-sensitivity C-reactive protein was associated with incident diabetic nephropathy in African Americans in the Jackson Heart Study [42]. Biomarker-based approaches have further highlighted the role of inflammation in the progression of kidney disease in subjects with DM by showing an association of an inflammatory risk signature with incident ESKD during 8-13 years of follow-up [43]. The detrimental combination of chronic tubulointerstitial inflammation and mitochondrial dysfunction ultimately leads to a profibrotic milieu that is responsible for irreversible kidney injury.

## 3. Anti-Inflammatory Effects of Novel Antidiabetic Agents

### 3.1. DPP4 Inhibitors

DPP4 or CD26 is a cell surface type II transmembrane protein which exerts noncatalytic functions by binding to contiguous proteins on the cell membrane or in the extracellular matrix [44,45]. It is expressed in a variety of cells, including immune, epithelial, and endothelial cells [44,45]. Its main role is the inactivation of incretins and the post-translational maturation of chemokines secreted by the activation of T cells [46,47]. DPP4is are well-known drugs for their antidiabetic effect. DPP4i was first identified as a therapeutic medication for T2DM because of its ability to upregulate the half-life of incretins, GLP-1, and glucose-dependent insulinotropic peptide (GIP). Generally, these incretins control blood glucose levels after a meal by promoting insulin release, prolonging gastric emptying, inducing satiety, suppressing glucagon release, and conserving beta-cell mass [46,47]. In cases of T2DM, administration of DPP4i increases glucose metabolism by increasing GLP-1 receptor signaling in the pancreas, which stimulates insulin production while suppressing glucagon secretion [48]. Sitagliptin constitutes the first drug from the category approved for the treatment of T2DM in 2006; since then, a variety of selective DPP4is have been introduced [47].

DPP4is, in addition to their antidiabetic effect, present benefits based on their anti-inflammatory action [45]. More particularly, DPP4 may play important roles in other processes other than glycemic control, as shown by its wide expression on several types of immune cells [49]. It is well known that DM constitutes a progressive low-grade inflammatory condition burdening patients with metabolic syndrome or an overall atherosclerotic profile. The wide use of DPP4i during the last two decades has raised awareness in the scientific community concerning the research of their anti-inflammatory effect [50,51]. DPP4is, due to their biochemical structure and enzymatic function, can activate or inactivate several fragments of the targeted chemokines [46]. The mechanisms underlying DPP4i’s protective effects include increased bioavailability of its substrates; effects on mediators and signaling pathways that improve cardiovascular and renal function by suppressing oxidative stress, inflammation, fibrosis, and apoptosis; improved endothelial function; and tissue reparation [52]. Based on this effect, alogliptin, a DPP4i, was shown to suppress toll-like receptor 4-mediated extracellular signal-regulated kinase, IL-6, and IL-1β secretion [53,54]. In addition, in vitro experiments of sitagliptin’s analogue to human macrophages showed inhibition of NF-κB p65 nuclear translocation through the cAMP/protein kinase A pathway, resulting in attenuation of proinflammatory status [55]. In this respect, a recent meta-analysis of 16 studies assessing the anti-inflammatory effect of DPP4i in patients with T2DM showed a significant reduction in C-reactive protein levels [56].

In animal experimental models, the use of sitagliptin led to a significant reduction in the levels of circulating proinflammatory cytokines such as IL-1β, IL-6, TNF-a, and monocyte chemoattractant protein (MCP)-1 [55]. Using sitagliptin analogue in various doses, a reduction was observed in the infiltration of CD8^+^ T cells and proinflammatory (M1) macrophages in the adipose tissue of diabetic mice, as well as improvement in their metabolic status [57]. Regarding the inflammatory burdening of atherosclerotic plaques, sitagliptin and alogliptin administration improved plaque stabilization and expression of IL-6/IL-1 in atherosclerotic lesions [54,58]. DPP4is suppress macrophage accumulation and foam cell formation reducing the total aortic atherosclerotic lesions in atherosclerotic mice [54,58]. In human umbilical vein endothelial cells and monocytes, linagliptin was reported to decrease IL-6 production [59]. Only one study showed no correlation between linagliptin administration and ferritin levels in 25 diabetes patients on hemodialysis, confirming the scarcity of data on humans [60].

In the clinical setting, DPP4 seems to be overexpressed in patients with T2DM, while cellular and serum DPP4 expression correlates well with glucose control [61]. In a cohort of diabetic patients treated with sitagliptin, a decrease in the levels of proinflammatory biomarkers such as CRP and IL-6 was observed [62]. Regarding the expression of DPP4 on peripheral blood monocytes, treatment with sitagliptin led to a reduction in DPP4 expression on their surface, while the unfavorable proinflammatory (M1)/anti-inflammatory (M2)-like phenotypes were improved [62]. The anti-inflammatory effect of DPP4i has also been recognized in patients with coronary artery disease and uncontrolled glucose levels. DPP4 seems to improve arterial stiffness measured via PWV as well as peripheral endothelial dysfunction [63]. Finally, in light of these complex effects of DPP4i, improvement in the inflammatory status of patients with sterile inflammatory diseases such as rheumatoid arthritis and inflammatory bowel disease have been documented [64,65].

### 3.2. GLP-1 Receptor Agonists

GLP-1 is a peptide hormone that presents 50% amino acid homology with glucagon. GLP-1 is mainly expressed by the intestinal L-cells as well as by brain cells after the degradation of proglucagon by prohormone convertase enzymes in response to nutrient ingestion [66,67]. Despite their high degree of sequence similarity, glucagon and GLP-1 have opposing effects on glucose homeostasis [68]. Glucagon causes a rise in glycemia, whereas GLP-1 causes a decrease in blood glucose levels. GLP-1’s hypoglycemic activity is linked to increased glucose-dependent insulin secretion, suppression of glucagon synthesis, and modulation of islet cell proliferation, differentiation, and survival. Furthermore, GLP-1 regulates satiety and decreases gastric emptying, which is consequently linked to weight reduction. As a result, GLP-1 has been extensively researched as a potential therapy for T2DM, while GLP-1 receptor antagonists and DPP4i are currently frequently used in the clinical setting [67,69].

GLP-1 receptor is widely expressed, suggesting that it may have additional roles other than glucose level regulation (Table 1). Evidence showed an anti-inflammatory effect on the cardiovascular system as well as on the pancreas, kidneys, and lungs [70]. In vitro studies revealed a downregulation in the expression of inflammatory genes such as NFκB1(p105), NFκB2(p100), RelA (also termed p65), TNF receptor superfamily member 1A, and receptor-interacting serine/threonine kinase 2 when treatment with a GLP-1 receptor agonist was applied in cultured human cells [71]. Exendin-4, a GLP-1 receptor antagonist, seems to suppress C-X-C motif chemokine 10 production, leading to decreased cytotoxic T lymphocyte cell recruitment in subjects with DM type I [72]. Another study showed that exenatide can induce the expression of serine protease inhibitor-9 in pancreatic islets, constituting a determining role in the survival of those cells against the attack from natural killers and cytotoxic T cells [73]. However, a hallmark in the pathophysiology of T2DM is the insulin resistance of adipose tissue. The main contributors to this phenomenon seem to be the infiltration of proinflammatory cytokines and chemokines in the tissue [74,75]. The administration of a recombinant adenovirus-producing GLP-1 to mice leads to the inhibition of NF-κB activation and phosphorylation of ERK1/2 and c-Jun N-terminal kinases. More particularly, the levels of common proinflammatory biomarkers (TNF-a, MCP-1, IL-6) were reduced [76]. As a result, in addition to glucose-lowering effects, GLP-1 receptor antagonists assist in the reduction in an individual’s inflammation grade, while also providing additional advantages such as islet preservation and potentially improved insulin sensitivity.

Regarding the anti-inflammatory properties of this drug category in the vascular bed, liraglutide seems to be the most widely studied. It was shown that administration of 100μM liraglutide resulted in a significant reduction in adhesion molecules’ gene expression [77,78]. More specifically, liraglutide’s anti-inflammatory effect is based on the activation of adenosine monophosphate-activated protein kinase (AMPK) and calcium/calmodulin-dependent protein kinase β (CAMKKβ), which are cAMP/Ca2+ signaling pathways [77,78]. Furthermore, an overexpression of sirtuin 6 (SIRT6) in endothelial cells and asymptomatic atherosclerotic plaques was observed. A SIRT6 level increase has been previously associated with the downregulation of proinflammatory genes expression [79,80]. The GLP-1 receptor’s beneficial effect extends to the reduction of oxidative stress. Indeed, the treatment of liraglutide to mice with high levels of catalase and glutathione peroxidase-3—important enzymes for regulating oxidative stress—increased their circulation levels [81,82]. A recent meta-analysis strongly supports the clinically relevant anti-inflammatory and antioxidant effects of GLP-1 receptor antagonists. An analysis of GLP-1 receptor antagonists versus standard diabetes therapies or placebo revealed significant reductions in CRP, TNFα, and malondialdehyde, and significant increases in adiponectin [83]. Finally, GLP-1 receptor antagonists seem to protect against contractile dysfunction in experimental animal models, as well as in diabetic cardiomyopathy [84,85]. Probably, GLP-1 receptor antagonists’ anti-inflammatory properties may be used to treat a wide range of conditions, including neurodegenerative brain disorders, kidney disease, nonalcoholic steatohepatitis, and pulmonary inflammatory diseases. However, certain uncommon incidents of acute pancreatitis and neoplasms have been recorded; consequently, the safety of GLP-1-based treatment should be evaluated in additional studies [86,87].

Probably, GLP-1 receptor antagonists’ anti-inflammatory properties may be used to treat a wide range of conditions, including neurodegenerative brain disorders, kidney disease, nonalcoholic steatohepatitis, and pulmonary inflammatory diseases. Regarding Alzheimer’s disease, recent studies illustrate that administration of liraglutide can reduce the inflammatory status in the cortex while restoration effects were observed [88,89]. Kim et al. highlighted the inhibitory effect of exendin-4 on microglial activation, suggesting that it may have therapeutic potential for the treatment of Parkinson’s disease [90]. Diabetic nephropathy is characterized by low-grade inflammation in the glomerular microvasculature of the kidney [91]. Studies showed that GLP-1-based treatments can reduce macrophage infiltration and inflammatory molecules in diabetic nephropathy models [92]. Anti-inflammatory efficiency has been proved not only in diabetic nephropathy but in nondiabetic as well. Generally, GLP-1-based treatments seem to reduce the infiltration of CD68-positive inflammatory macrophages in the kidney [93]. In individuals with nonalcoholic fatty liver disease, GLP-1 receptor agonists reduced alanine aminotransferase and aspartate aminotransferase levels, improving lipid metabolism [94]. In addition, a significant reduction in CRP and TNF-a circulating levels was observed in this special patient population [95,96]. However, certain uncommon incidents of acute pancreatitis and neoplasms have been recorded; consequently, the safety of GLP-1-based treatment should be evaluated in additional studies [86,87].

### 3.3. SGLT2-Inhibitors

Because they are responsible for the reabsorption of the bulk of filtered glucose by the glomeruli, SGLT1 and SGLT2, which are found on the proximal convoluted tubule of nephrons, are essential mediators of glucose homeostasis. The high-capacity, low-affinity SGLT2 absorbs 90% of the glucose, with a sodium turnover ratio of 1:1 [97,98]. As a result, facilitative glucose transporters (GLUTs) situated in the epithelial lining of proximal tubules unleash glucose into the blood [97]. Overexpression of glucose transporter genes in diabetic individuals has been described, leading to upregulation of SGLT2 and consequently enhanced reabsorption [99]. The particular processes involved are unknown, although they appear to be linked to the underlying hyperglycemia [99].

The impact of SGLT inhibition on diabetes was first observed in 1987 with the use of phlorizin, a nonspecific SLGT1 and SGLT2 inhibitor [100]. Its treatment resulted in reduced hyperglycemia and increased glycosuria in an animal model of T2DM, along with normal insulin sensitivity. However, there were intrinsic limits that had to be solved. The simultaneous inhibition of SGLT1 on the intestinal mucosa resulted in diarrhea [101], whereas phlorizin’s hydrolysis to phloretin, which inhibits GLUT1, might result in poor glucose uptake in numerous tissues [102]. However, specific SGLT2 inhibitors have yet to be produced. Dapagliflozin [103] was the first high-potency SLGT2-i to be created, followed by canagliflozin [104], empagliflozin [105], and ertugliflozin [106]. Other compounds of this family, such as ipragliflozin, tofogliflozin, and luseogliflozin, have also been explored, but less thoroughly. In terms of hypoglycemic impact, all licensed medications have shown efficacy in decreasing glucose levels and HbA1c in randomized clinical studies.

Despite being first used to treat T2DM [107], SGLT2 inhibition has been at the center of scientific research in recent years due to its favorable cardiorenal profile. Recently completed clinical trials have shown significant improvements in individuals with heart failure and chronic kidney disease (CKD) [108,109,110,111]. Notably, the results were similar regardless of the presence of DM [112]. Furthermore, SGLT2 inhibitors have emerged lately as the sole pharmacologic treatment with proven effectiveness in individuals with heart failure and retained ejection fraction [113]. It should also be stressed that various additional offset effects, such as a lower incidence rate of atrial fibrillation and an improvement in nonalcoholic liver disease indices after therapy with an SGLT2 inhibitor, have been documented [114,115].

Their pleiotropic mechanism of action is believed to be accountable for these beneficial actions, including the management of inflammation (Table 2). In fact, inflammation could be contributing to incident endothelial dysfunction, oxidative stress, and platelet activation, among others [33,116]. However, clinical evidence of their impact on these parameters, notably inflammation, is sparse. SGLT2 inhibitors may reduce levels of inflammatory markers such as CRP, IL1, IL-6, and TNF-α in the few cohort studies that have been published [117,118,119,120]. Furthermore, a 6-month therapy with dapagliflozin 5 mg once daily resulted in a substantial decrease in plasma TNF-α in a small-scale RCT of 35 patients with T2DM and coronary artery disease [121].

A recent hypothesis for the probable mechanism involved in this anti-inflammatory action includes the restoration of autophagy, which leads to antiapoptotic, antioxidant, and anti-inflammatory effects. In this context, SGLT2 inhibitors may induce a state of perceived energy deprivation, leading to an increase in the autophagy-stimulating SIRT1/hypoxia-inducible factor/AMPK pathway [130], while decreasing the autophagy-inhibiting Akt/mammalian target of rapamycin complex 1 (mTORC1) pathway [131]. As a result, restoring autophagy may mitigate the aforementioned negative effects, including inflammation. 

The anti-inflammatory effect of SGLT2 inhibitors has also been assessed in preclinical models. Beginning with diabetic animal models, several contemporary studies have pointed towards an anti-inflammatory effect, evidenced by the lowering of inflammatory marker expression (MCP-1, IL-6, p38) [122,123,124]. In models of CKD, empagliflozin ameliorated IL-1β-induced inflammation in normoglycemic human proximal tubular cells, indicating a glucose-independent anti-inflammatory action of SGLT2 inhibitors through targeting *CXCL8/IL8*, *LOX*, *NOV*, *PTX3*, and *SGK1* genes [132]. The SGLT2 inhibitor dapagliflozin resulted in attenuation of renal inflammation and apoptosis in a diabetic rat model [125]. Moreover, dapagliflozin led to itaconate-mediated NLRP3 inflammasome downregulation in an animal model of progressive CKD [133]. Empagliflozin diminished the renal expression of inflammatory markers (TNF-α, MCP-1) by acting on the HMGB1-TLR4 axis in an experiment involving rats with streptozocin-induced diabetes [126]. These findings may partly explain the attenuated glomerular and tubulointerstitial injury observed in CKD following treatment with an SGLT2 inhibitor [134]. In models of cardiovascular disease, Quaqliarriello et al. demonstrated the anti-inflammatory potential of empagliflozin in an animal model of doxorubicin-induced cardiotoxicity through the downregulation of NF-κB, NLRP3 inflammasome, and inflammatory interleukins. Empagliflozin also attenuated interleukin-17A-induced human aortic smooth muscle cell proliferation and migration by targeting TRAF3IP2/ROS/NLRP3/Caspase-1-dependent IL-1beta and IL-18 secretion [135]. SGLT2 inhibitors may exert anti-inflammatory effects in nonalcoholic steatohepatitis, since ipragliflozin and empagliflozin administration reduced lobular inflammation and inflammatory marker expression [128,129,136]. Lastly, recent studies have documented the anti-inflammatory potential of dapagliflozin in animal models of experimental colitis through inhibition of NADPH oxidase 2 and the NF-κB/AMPK/NLRP3 axis, among others [137,138].

## 4. Future Directions

The scientific interest in the management of not only T2DM but also obesity via the use of GLP-1 receptor agonist and the older one, glucose-dependent insulinotropic polypeptide (GIP), led to the development of newer agents [139,140,141]. The dual GIP/GLP-1 receptor agonist has outperformed dulaglutide and semaglutide, two highly selective GLP-1 receptor agonists, in terms of lowering plasma glucose and glycated hemoglobin (HbA1c). Until now, the main representative of this drug category has been Tirzepatide, which combines the antiglycemic benefits of both, augmenting insulin secretion [142]. However, in previous studies on diabetic patients, GIP did not influence the overall effect of the combination in type 2 diabetes patients. Rather, the combination of GIP and GLP-1 was less insulinotropic than the sum of the individual effects of GIP and GLP-1 given separately [142]. A potential explanation could be provided by the GIP resistance that diabetic patients present. Recently, the antiatherosclerotic effect of GIP, reducing oxidative stress in human endothelial cells and inflammatory cytokine release in visceral adipose tissue, was shown [143,144]. Moreover, the latest evidence suggests a significant improvement in glycemic control and obesity with tirzepatide compared to placebo, GLP1 receptor agonists, and insulin [145,146,147,148]. In addition, tirzepatide could ameliorate liver fat compared to insulin degludec, as recently shown in the SURPASS-3 MRI substudy [149]. The available data are sparse regarding the anti-inflammatory effect of GIP. Tirzepatide could induce reductions in hsCRP and ICAM-1 within 4 weeks of treatment in patients with T2DM [150]. According to the above-mentioned findings, it could be assumed that there is a possible synergistic anti-inflammatory role of dual GIP/GLP-1 receptor agonists, which has to be validated in future studies.

Subjects with and without T2DM benefit from SGLT2 inhibitors in terms of cardiac and renal outcomes. SGLT1 transporters are expressed in the intestines, kidney, brain, heart, trachea, testis, and prostate gland [151,152]. They control glucose absorption in the small intestine as well as the reabsorption of roughly 10% of filtered glucose in the upper section of the renal proximal tubule [151,152]. The development of dual SGLT1/SGLT2 inhibitors was a challenge for physicians trying to combine the benefits provided by the two substances [153]. Sotagliflozin, a first-in-class dual SGLT1 and SGLT2 inhibitor, has recently been evaluated in two trials of patients with T2DM: the Effect of Sotagliflozin on Cardiovascular and Renal Events in Patients With Type 2 Diabetes and Moderate Renal Impairment Who Are at Cardiovascular Risk (SCORED) trial and the Effect of Sotagliflozin on Cardiovascular Events in Patients With Type 2 Diabetes Post Worsening HF (SOLOIST-WHF) trial [154,155]. A recent meta-analysis showed the efficiency of sotagliflozin in terms of decreasing the risk of all-cause mortality, cardiovascular mortality, and hospitalization for heart failure, myocardial infarction, and kidney disease progression in patients with T2DM [156,157]. At present, however, no studies have been performed comparing the anti-inflammatory potential of dual SGLT1/SGLT2 inhibitors to highly selective SGLT2 inhibitors. Therefore, there is a need for both preclinical and clinical evidence to determine whether the combined inhibition of SGLT1/SGLT2 may promote more potent anti-inflammatory actions.

## 5. Conclusions

Inflammation appears to be the main contributor in the development of a variety of comorbidities. It is well known that DM is a multifactorial disease consisting of complex pathophysiological pathways driven by low-grade inflammation. Currently, antidiabetic drugs are at the forefront of scientific research, not only for their glucose control effect but also their anti-inflammatory potential. In this review, we highlighted the anti-inflammatory effects of widely administered antidiabetic drugs and we presented their importance in mitigating an individual’s inflammatory status. Moreover, we foresee their potential application in other chronic sterile inflammatory diseases, whether or not DM is present.

## Figures and Tables

**Table 1:** Anti-inflammatory effects of glucagon-like peptide-1 (GLP-1)-based drugs.

GLP-1-Based Drugs	Substance	Disease/Organ Target	Main Effect
GLP-1 Receptor Agonists	Exenatide Liraglutide Lixisenatide Albiglutide Taspoglutide Dulaglutide	Diabetes Atherosclerosis–Cardiovascular disease Asthma Alzheimer’s disease Parkinson’s disease Nonalcoholic Steatohepatitis (NASH) Nephropathy Testis Psoriasis	▪ Levels of CRP, TNF-α, IL-1β, IL-6, MCP-1, and soluble adhesion molecules ▪ Suppress C-X-C motif chemokine 10 production ▪ Inhibit of nuclear factor-kappa B (NF-κB) ▪ Increase of SIRT6 levels
DPP4 Inhibitors	Sitagliptin Des-fluoro-sitagliptin Alogliptin Linagliptin Vildagliptin NVP-DPP728 Anagliptin Saxagliptin	Diabetes Vascular Disease Nephropathy Neurodegenerative brain disorder	▪ Levels of IL-1β, IL-6, MCP-1, and soluble Adhesion Molecules ▪ Suppress toll-like receptor 4 mediated extracellular signal-regulated kinase ▪ Inhibition of NF-kappaB p65 nuclear translocation ▪ Improve arterial stiffness and peripheral endothelial dysfunction

DPP4: dipeptyl peptidase-4, CRP: C-reactive protein, IL: interleukin, MCP-1: monocyte chemoattractant protein-1, TNF-a: tumor necrosis factor alpha, SIRT6: sirtuin 6.

**Table 2:** Selected preclinical studies demonstrating the anti-inflammatory effects of sodium-glucose cotransporter-2 (SGLT2) inhibitors.

Study	Preclinical Model	Disease	SGLT2 Inhibitor	Finding
Madonna et al. [122]	Male C57BL/6 mice	DM	Empagliflozin	↓ p38
Ganbaatar et al. [123]	Male ApoE/mice	DM	Empagliflozin	↓ aortic expression of MCP-1, VCAM-1, NOX2 and p22phox ↓ PVAT expression of MCP-1, ICAM-1, VCAM-1, CD68, p47phox, and p22phox
Radlinger et al. [124]	Male db/db mice	DM	Empagliflozin	↓ cardiac IL-6, CCL2, CCR2 ↑ cardiac IL-10
Elkazzaz et al. [125]	Male albino rats	DM	Dapagliflozin	↑ PTX-3 ↓ TNF-α
Ashrafi Jigheh et al. [126]	Male Wistar rats	DM	Empagliflozin	↓ NF-κB in renal cortex ↓ renal TNF-α and MCP-1 ↓ urinary IL-6
Quaqliariello et al. [127]	Female C57Bl/6 mice	Doxorubicin-induced cardiotoxicity	Empagliflozin	↓ IL-6, IL-8, NF-κB, NLRP3
Perakakis et al. [128]	Male C57BL/6JRj mice	NASH	Empagliflozin	↓ hepatic lobular inflammation
Tahara et al. [129]	KK/A^y^ mice	DM + NASH	Ipragliflozin	↓ plasma and liver IL-6, MCP-1, TNF-α, and CRP

MCP-1: monocyte chemoattractant protein-1, VCAM-1: vascular cell adhesion molecule-1, NOX2: NADPH oxidase 2, ICAM-1: intercellular adhesion molecule-1, IL: interleukin, CCL2: chemokine ligand 2, CCR2: chemokine receptor 2, PTX-3: pentraxin-3, TNF-α: tumor necrosis factor-alpha, A^y^: lethal yellow, CRP: C-reactive protein.

## Data Availability

Not applicable.
